# Relationship between gaming disorder across various dimensions among PUBG players: a machine learning-based cross-sectional study

**DOI:** 10.3389/fpsyt.2023.1290206

**Published:** 2023-12-18

**Authors:** Ali Hassan, Muhammad Shahzad, Muhammad Daniyal, Wael Hafez, Syed Fahad Javaid, Moien AB Khan

**Affiliations:** ^1^Department of Media and Communication Studies, The Islamia University of Bahawalpur, Bahawalpur, Pakistan; ^2^Department of Statistics, Faculty of Computing, The Islamia University of Bahawalpur, Bahawalpur, Pakistan; ^3^Internal Medicine Department, The Medical Research Division, The National Research Center, Cairo, Egypt; ^4^Health and Wellness Research Group, Department of Psychiatry and Behavioral Sciences, College of Medicine and Health Sciences, United Arab Emirates University, Al-Ain, United Arab Emirates; ^5^Health and Wellness Research Group, Department of Family Medicine, College of Medicine and Health Sciences, United Arab Emirates University, Al-Ain, United Arab Emirates

**Keywords:** addiction, gaming disorder, cultivation effect, machine learning approach, behavior, health and wellness, religion

## Abstract

**Background:**

PlayerUnknown’s battlegrounds (PUBG), a widely played multiplayer online game, has sparked interest and concern regarding its impact on players. This study explored the relationship between factors such as cultivation level, motivation, religious engagement, gaming disorder, and addiction among PUBG players.

**Methods:**

This study employed a convenience sampling technique to select a sample of 500 PUBG players. An Artificial Neural Network (ANN) model was used to identify the primary factors contributing to the level of cultivation.

**Results:**

Male participants exhibited a higher level of cultivation than their female counterparts did. According to the ANN model, gaming disorder exhibited the greatest normalized importance, with a value of 100%. This was followed by the religious level, which had a normalized importance of 54.6%. Additionally, motivation level and gaming addiction demonstrated normalized importance values of 47.6 and 44.4%, respectively. This study revealed a statistically significant correlation between engaging in PUBG and the cultivation effect observed among respondents.

**Conclusion:**

This study highlights several noteworthy factors, including gaming disorder, religious affiliation, motivation level, and gaming addiction. These factors offer valuable insights into understanding gaming behavior and devising effective interventions.

## 1 Background

The advent of digital gaming, specifically online multiplayer games, has had a significant and far-reaching influence on society, leading to a fundamental shift in the entertainment realm (1). PlayerUnknown’s Battlegrounds (PUBG) is distinguished within the domain of gaming because of its captivating gameplay and compelling attributes (2). PUBG Corporation released PUBG Mobile in 2018, which is free to play. PUBG Mobile had more than 1.1 billion downloads worldwide and earned $ 9 billion in revenue as of December 2022 (3). Nevertheless, the growing prevalence of gaming has prompted apprehensions regarding the adverse consequences of excessive gaming, encompassing gaming addiction and disorders (4).

The World Health Organization (WHO) has officially acknowledged gaming disorder as a mental health disorder in its International Classification of Diseases (11th edition, 2018). The WHO defines gaming disorder as consistent and repetitive engagement in gaming activities that supersedes other personal interests and activities (5). The diagnostic criteria for gaming disorder encompass three key elements: compromised regulation of gaming behavior, elevation of gaming as a primary focus over other pursuits, and persistent continuation of gaming despite experiencing unfavorable outcomes (6). The addictive nature of PUBG has been widely recognized, as evidenced by the substantial time commitment players devote to the game and their consequent neglect of crucial life domains (7). The phenomenon of gaming addiction has been found to exert negative consequences on individuals’ overall well-being, encompassing adverse impacts on their academic performance and physical health issues (8, 9). Psychopathological conditions, including anxiety, depression, and social withdrawal, can arise because of maladaptive gaming behaviors (10, 11). It is imperative to critically examine the process by which gaming behaviors are pathologized and to differentiate between authentic disorders and highly dedicated hobbies (12).

Cultivation theory, a fundamental concept in media studies, asserts that extensive engagement with media can influence individuals’ cognitive frameworks and moral principles. Cultivation theory can be effectively employed in the context of video games, particularly because of the escalating level of realism that closely resembles real-world experiences. Prolonged exposure to gaming has the potential to shape individuals’ perceptions of reality and their assimilation of values and beliefs prevalent within the game (13). Nevertheless, critics have contended that this particular viewpoint fails to acknowledge the individual agency and critical thinking abilities of gamers, as it presupposes a passive inclination towards media messages (14). The application of cultivation theory allows for an examination of the potential impacts of prolonged engagement with PUBG on individuals’ cognitive processes, moral values, and behaviors. It is imperative to thoroughly examine the underlying assumption that players internalize in-game values, as there is potential for a distinction between the virtual realm and actuality (15).

The impact of media influence is affected by an individual’s level of motivation, as demonstrated by Martens and Hobbs (16). Their research suggested that individuals with high levels of motivation are less likely to be susceptible to the effects of media (16). Motivation is a multifaceted phenomenon that is subject to the influence of various internal and external factors (16, 17). The phenomenon of religiosity, defined as the degree of adherence to religious beliefs and practices, has been observed to correlate with media influence and cultivation theory (18). According to Ahmadi and Saghafi (19), there is evidence to suggest that individuals with higher levels of religiosity may experience a reduced risk of developing gaming addiction and disorder (19). In addition, religiosity has been associated with reduced Internet and smartphone usage (20). This finding highlights the potential impact of religious beliefs on gaming behavior.

In recent years, online gaming has witnessed a significant surge in popularity. Games, such as PUBG, have captured the attention of millions. Despite its appeal, there has been no comprehensive research on the various effects of gaming disorders, especially when it comes to PUBG. Existing studies have focused on gaming disorders in general and have not delved into the unique aspects of popular games, such as PUBG (21). This research gap is important because understanding the dynamics of gaming disorders related to played games is crucial for developing effective interventions and policies. This study aimed to bridge this gap by conducting an analysis using machine learning techniques to explore gaming disorders across dimensions among PUBG players. This study aims to provide insights into the patterns, motivations, and impacts of gaming disorders within this globally popular game, contributing significantly to the existing literature. This study investigated the impact of gaming disorder, religious beliefs, addiction, and motivation levels on PUBG players’ cultivation levels, filling a gap in the current research. While the literature extensively explores gaming disorders and addiction, this study uniquely incorporates religious beliefs and motivations, offering a holistic view of the factors influencing gaming behavior. By examining these variables, this study aims to understand the complex interplay between personal and cultural influences on gaming, particularly in the context of PUBG, a game that significantly impacts youth engagement globally. Thus, the primary goal of this research was to investigate the underlying motivations and consequent impacts of PUBG on behavioral patterns among gamers. This study adds to the body of knowledge by illuminating the complex facets of gaming behavior. As a result, this study lays the groundwork for developing comprehensive prevention and intervention strategies.

## 2 Materials and methods

### 2.1 Study design and sample size calculation

This cross-sectional study investigated the relationship between PUBG gaming and its impact on cultivation effect, religious level, motivation, gaming disorder, and Internet gaming disorder. The required sample size was estimated using the Raosoft sample size calculator (22) with a margin of error of 5% and a confidence level of 95%. The calculated sample size was determined to be 377 respondents.

### 2.2 Data collection

Data collection for this study was conducted between September 21, 2021, and November 22, 2021. Participants in this study were recruited using a convenient sampling method. The initial step in the recruitment process involved identifying potential participants who were active players in PUBG on mobile devices. To ascertain this, individuals were approached with a preliminary screening question: ‘Do you play the PUBG game on your cell phone?’ Only those who affirmed their engagement in playing PUBG on their mobile devices were considered eligible and subsequently selected for participation in the study. This approach ensured that the study sample consisted exclusively of active PUBG players, aligning with the specific focus of our research.

### 2.3 Inclusion and exclusion criteria

The study included both male and female respondents who played PUBG on their cell phones and were proficient in English. The exclusion criteria encompassed Individuals who did not play the PUBG game on their cell phones, had been diagnosed with mental health illnesses, or declined to answer further survey questions were excluded.

### 2.4 Questionnaire design

The survey consisted of multiple sections. Initially, data pertaining to demographic attributes including gender, age, marital status, and educational attainment were gathered. Furthermore, the duration of engaging in PUBG on mobile devices was evaluated. Various scales were incorporated to assess the distinct variables, as described below.

Cultivation theory, a key concept in the fields of communication and media studies, examines how prolonged exposure to media, especially television, influences an individual’s perception of reality. This theory suggests that consistent exposure to specific themes and portrayals in the media can mold an individual’s worldview, impacting their beliefs and attitudes over time (23). Cultivation Theory, which was initially employed to analyze the impact of conventional media such as television, has been utilized to evaluate the cultivation effect of video gaming (23). The participants were classified into three tiers based on their level of engagement: low cultivation (playing for less than 2 h), medium cultivation (playing between 2 and 4 h), and high cultivation (playing for more than 4 h) (24).

The Abraham Religious Scale (ARS) is a measurement tool comprising 10 items that assess an individual’s religious level. Respondents were asked to rate their agreement with each item on a 3-point Likert scale, with options ranging from 1 (agree) to 3 (disagree). According to Khodayarifard et al. (25), there is an inverse relationship between higher scores and religious status (25).

The Motivation Level Scale was developed to evaluate individuals’ motivations for engaging in video gameplay, drawing from the theoretical framework of uses and gratification theory. This scale encompasses various factors such as enthrallment, challenge, competition, social interaction, recreation, and fantasy (26). The scale consists of 20 questions evaluated using a 5-point Likert scale, with response options ranging from 1 (Strongly Agree) to 5 (Strongly Disagree). The scores range from 20 to 100, with lower scores indicating diminished levels of motivation for gaming.

The Gaming Addiction Scale (GAS) was used to assess the degree of problematic gaming behavior. The GAS comprises a set of seven items, where higher scores are indicative of a higher level of addiction to gaming. The participants were asked to rate the frequency of their gaming activities on a five-point Likert scale, with response options ranging from 1 (never) to 5 (very frequently) (27).

The Internet Gaming Disorder Scale 9—Short Form (IGDS9-SF) was used to evaluate the severity of symptoms related to Internet gaming disorder (IGD). This scale employed a 5-point Likert scale, with responses ranging from 1 (never) to 5 (very often) (28). The scale comprises a total of nine items, with higher scores indicating a greater degree of problematic Internet gaming.

### 2.5 Neural networking model for predicting cultivation effect

A neural network model is a machine-learning model that can be used to estimate the relationship between dependent and independent variables. In a neural network model, data are inputted into a network of interconnected nodes (also called neurons) organized into layers. The first layer receives the input data, and the last layer outputs the prediction of the model. Between, there can be any number of hidden layers that process the data and transform it in a manner that helps the model make better predictions. During training, the neural network adjusts the weights and biases of the connections between neurons based on the input data and the desired output. This process of adjusting the weights and biases is called backpropagation. Neural networks can be used for various tasks, including regression (estimating a continuous dependent variable) and classification (estimating a categorical dependent variable). In a regression neural network, the output of the model is a continuous value that represents the predicted value of a dependent variable. The input to the model can be one or more independent variables, and the hidden layers transform the input data into predictions. To perform neural network investigations, motivation level, religious level, gaming addiction, and gaming disorder were included as independent variables. The dependent variable was the level of time spent playing PUBG. The Statistical Package for Social Sciences (SPSS) version 27 was utilized as a software tool to build a neural network model with precision.

### 2.6 Ethical considerations

This study adhered to the principles outlined in the Declaration of Helsinki for recruitment of human subjects. Prior to participation, each respondent received a brief explanation of the study objectives and provided informed consent. The Strengthening the Reporting of Observational Studies in Epidemiology (STROBE) guidelines were followed to report this study (29).

### 2.7 Statistical analysis

The reliability of the questionnaire was evaluated using Cronbach’s alpha, a statistical measure that assesses the internal consistency of the instrument. The normality of the data was assessed using the Kolmogorov–Smirnov test to verify compliance with the underlying assumptions of the statistical tests. Descriptive statistics, such as mean age, median hours spent playing PUBG, and percentage of responses, were used to describe the demographic and religious levels, motivation levels, gaming disorder, and gaming addiction of the participants. The statistics presented in this study offer a comprehensive depiction of the sample and the data distribution.

The present study employed *χ*^2^-analysis to examine the relationship between the duration of engagement in PUBG and various factors including demographic and religious characteristics, motivation level, gaming disorder, and gaming addiction. Furthermore, a neural network, a type of supervised machine-learning model, was employed to evaluate the influence of different study factors on the duration of engagement in playing PUBG. Neural networks have demonstrated efficacy in discerning intricate patterns and correlations within intricate datasets, and are frequently employed to forecast a dependent variable by considering numerous independent variables. The application of this machine-learning approach facilitated the assessment of the relative importance of each predictor variable in determining the impact of cultivation on users of the PUBG game.

Statistical analyses were conducted using SPSS version 27. Statistical significance was assessed at a significance level of 0.05, indicating that the observed outcomes were improbable to have arisen because of random chance.

## 3 Results

This study demonstrated a high level of reliability, with a Cronbach’s alpha (α) of 0.870. Out Of the 500 participants, 82% (*n* = 410) were male and 18% (*n* = 90) were female. The findings indicated that males showed a greater inclination towards playing online PUBG games than females. The average age of the respondents in the study was 19.94 ± 1.14 years (Table 1).

**Table 1:** General attributes of the respondents with significance.

Study variables	Low cultivation effect (LCE)	Medium cultivation effect (MCE)	High cultivation effect (HCE)	Test-value	*p*-value
Age	19.77 ± 1.086	20.04 ± 1.195	19.97 ± 1.145	0.856	0.425
Gender				6.532	0.038 < 0.05
Male	49 (9.8)	50 (10)	311 (62.2)		
Female	4 (0.8)	7 (1.4)	79 (15.8)		
Area of living				1.407	0.495
Urban	35 (7)	37 (7.4)	231 (46.2)		
Rural	18 (3.6)	20 (4)	159 (31.8)		
Education level				11.9	0.064 < 0.10
Under matriculation	42 (8.4)	39 (7.8)	238 (47.6)		
Matriculation	6 (1.2)	4 (0.8)	76 (15.2)		
Undergraduate	3 (0.6)	8 (1.6)	42 (8.4)		
Graduate	2 (0.4)	6 (1.2)	34 (6.8)		
ARS				8.359	0.015 < 0.05
Low ARS level	21 (4.2)	33 (6.6)	236 (47.2)		
High ARS level	32 (6.4)	24 (4.8)	154 (30.8)		
Motivation level				9.099	0.059 < 0.10
Low motivation level	31 (6.2)	31 (6.2)	195 (39)		
Medium motivation level	15 (3)	20 (4)	99 (19.8)		
High motivation Level	7 (1.4)	6 (1.2)	96 (19.2)		
GAS				4.003	0.135
Low GAS level	24 (4.8)	34 (6.8)	178 (35.6)		
High GAS level	29 (5.8)	23 (4.6)	212 (42.4)		
IGD				8.447	0.015 < 0.05
Low IGD level	19 (3.8)	34 (6.8)	158 (31.6)		
High IGD level	34 (6.8)	23 (4.6)	232 (46.4)		

Table summarizes the characteristics of the respondents with respect to their cultivation effect, as follows: 1 = low, 2 = medium, and 3 = high. One-way analysis of variance (ANOVA) was used to compare the mean age of the three groups (LCE, MCE, and HCE). For the comparison of qualitative variables, the *χ*^2^-test of independence was applied, and p-values were adjusted for cells with scores less than 5. The ARS, GAS, and IGD levels were computed using median threshold values. 5 and 10% significance level has been chosen for comparison with p value.

The average age of respondents with low cultivation effect (LCE) was 19.77 ± 1.086, medium cultivation effect (MCE) was 20.04 ± 1.195, and high cultivation effect (HCE) was 19.97 ± 1.145. However, there was no significant difference in the average age among the different cultivation effect levels, indicating that age does not play a significant role in determining the low, medium, or high cultivation effects.

In terms of gender, male respondents were more likely to be in the HCE category. Among male respondents, 62.2% (*n* = 311) were categorized as HCE, while only 15.8% (*n* = 79) of female respondents were in the HCE category. Statistical analysis confirmed a significant association between gender and cultivation effects. The area of residence was not significantly associated with the cultivation effect. Among the respondents, 46.2% (*n* = 231) from urban areas were considered on the HCE scale, while 31.8% (*n* = 159) from rural areas were on the HCE scale.

When considering education level, 47.6% (*n* = 238) of respondents did not have matriculation qualification. 15.2% (*n* = 76) of those who had matriculated and only 6.8% (*n* = 34) of graduates were classified as heavy users. Although the educational level did not show a significant association with the cultivation effect, there was a significant association at the 10% level of significance.

Among the users who were HCE, 47.2% (*n* = 236) showed a low level of ARS. The association between ARS and cultivation effect was statistically significant (*χ*^2^ = 8.359, *p* = 0. 015). Regarding the motivation level of the respondents, 39% (*n* = 195) of the HCE users showed a low level of motivation, while 19.8% (*n* = 99) showed a median level, and 19.2% (*n* = 96) showed a high level of motivation. The association between motivation level and cultivation effect was significant at the 10% significance level. GAS levels were not significantly associated with the cultivation effect. Only 4.8% (*n* = 24) of the light users showed a low GAS level, while 35.6% (*n* = 178) of the heavy users showed a low GAS level, and 42.4% (*n* = 212) of the heavy users showed a high GAS level. Concerning Internet Gaming Disorder (IGD) levels, a notable significant association was observed with the cultivation effect. Among the respondents, 31.6% (*n* = 158) with high cultivation effect had a low IGD level, while 46.4% (*n* = 232) of heavy users had a high IGD level.

### 3.1 Correlation analysis of the study factors

**Figure 1:**
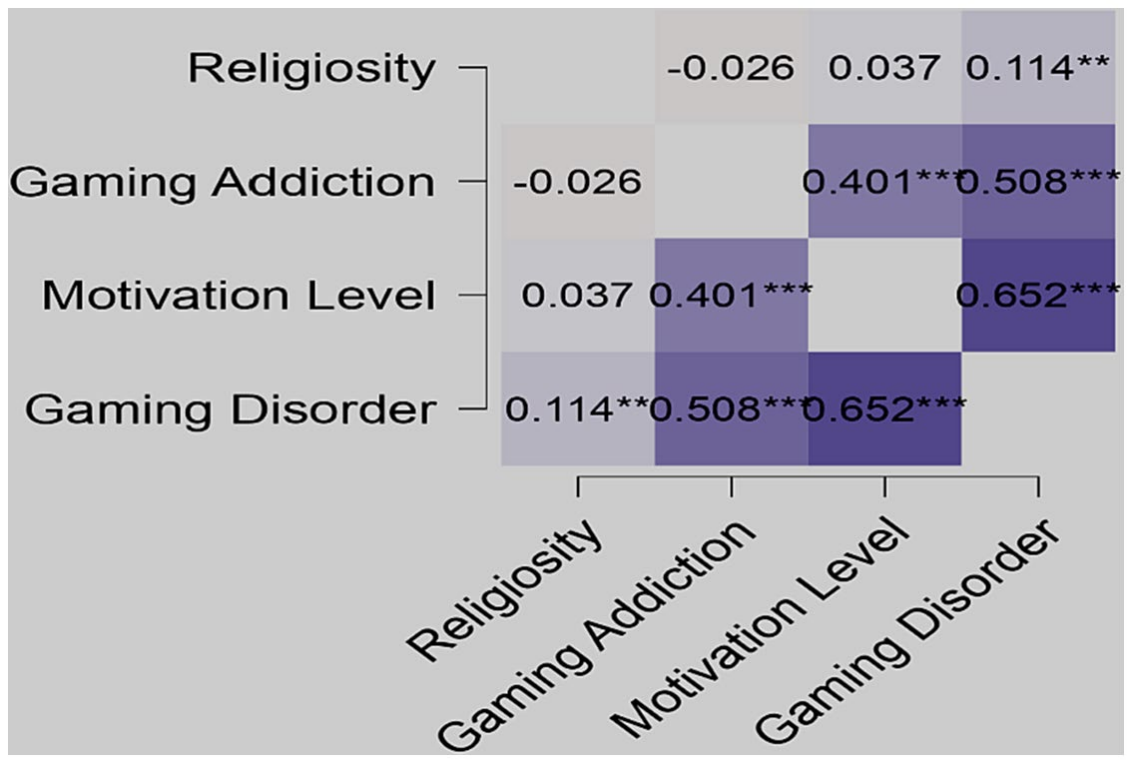
Correlation Heatmap of the significant ARS, GAS, ML, and IGD Levels. **Significant and ***Highly significant.

Spearman’s rank correlation was used to determine significant correlations among the factors investigated in this study that were associated with the amount of time spent on PUBG gaming. The factors examined were motivation level, religious level, gaming disorder, and gaming addiction. Figure 1 shows a Heatmap of the study factors with the significance highlighted.

Our findings revealed a weak significant correlation between religion level and gaming disorder (r = 0.114, 0.026**), indicating that religiosity has little impact on PUBG gameplay duration. On the other hand, we observed a significant positive correlation between gaming addiction and motivation level (r = 0.401, *p* = 0.000***), indicating that individuals with higher levels of gaming addiction were more motivated to play PUBG. Furthermore, our study showed a strong positive correlation between gaming addiction and gaming disorders (r = 0.508, *p* = 0.001***), indicating that individuals with higher levels of gaming addiction are more likely to develop gaming disorders. Interestingly, we also found a significant positive correlation between motivation level and gaming disorder (r = 0.652, *p* = 0.000), suggesting that individuals highly motivated to play PUBG are more likely to develop gaming disorder. Overall, our study highlights that various factors associated with PUBG gameplay duration are positively correlated, resulting in the association of factors that can influence an individual’s gaming behavior.

### 3.2 Parameter description for ANN model for cultivation effect

**Figure 2:**
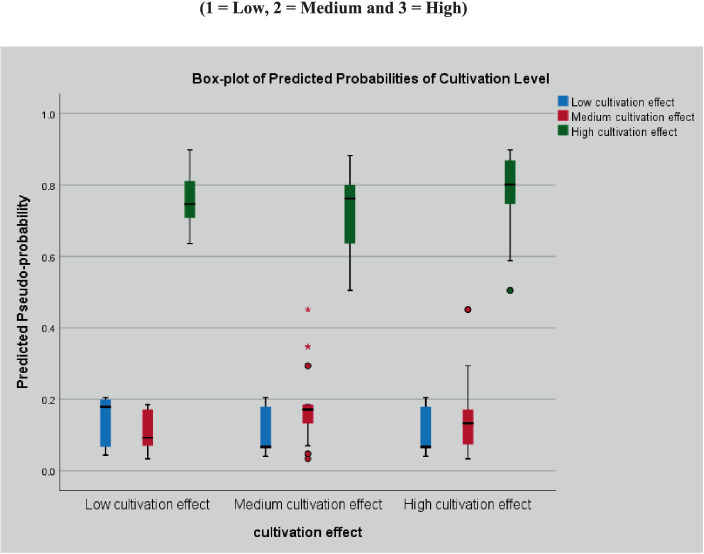
Boxplot of predicted probabilities associated with levels of cultivation effect (1 = Low, 2 = Medium, and 3 = High). The ANN model’s predicted pseudo-probability is presented by a box-plot diagram for the three cultivation level variable categories (1 = low, 2 = medium, and 3 = high). The blue box plot shows the low, red box plot shows the medium, and green box plot shows the predicted probabilities.

To achieve an optimal performance, it is crucial to establish a suitable neural network structure that includes a suitable number of neurons and hidden layers. The number of neurons must be carefully considered, as an excessive number can lead to over-fitting, while too few neurons may not be sufficient for processing the data. Therefore, multilayer perceptron (MLP) neural networks were chosen for data analysis. A backpropagation learning algorithm was used to train these networks, and gradient descent was used to update the weights, gradually reducing the error function. Seventy percent of the data was used to train the model, and 30% was used to test the ANN model. The dataset is divided into three parts. In this analysis, we looked at the response data to determine whether the MLP neural network could identify important predictors. The error function that the ANN model minimized during the training phase was the cross-entropy error, which was used for both the training and testing samples. The percentages of inappropriate predictions for training and testing samples were 22.3 and 19.5%, respectively. This shows that the ANN model made inaccurate predictions about the dependent variable (Cultivation Effect) for approximately 22.3% of the training and 19.5% of the testing data, which is adequate for further modeling. The automatic architecture was used in the design of the ANN model, as shown in Figure 2, which had three nodes for the computation of the hidden layer and three nodes for the output layer, which were used to categorize the results of the cultivation effect on the dependent variable. Various functions were utilized for various layers; The SoftMax function was used for the output layer, and the activation function served as the hyperbolic tangent of the hidden layer (Appendix 1). Subsequently, a box plot was developed to show the predicted probability of the observed level of cultivation effect categorized into low, medium, and high cultivation effects, as shown in Figure 2. The first box plot on the left indicates low cultivation effect. In the low cultivation effect category, the predictive probability of the high cultivation effect was close to 0.8, whereas the low and medium cultivation effects were close to 0.2 and 0.0. Similarly, for the selected ANN model, the predictive probability for the high cultivation effect in medium and high cultivation effects was also close to 0.8, while the predictive probability for both the low and medium cultivation effects was between 0 and 0.2. This shows that the predictive probability for a high cultivation effect of the respondents was higher than that of the respondents who had low and medium cultivation effects.

The ANN model was validated by the receiver operating characteristic (ROC) curve, which showed the classification performance for all possible cutoffs using a diagram of sensitivity and specificity. Table 2 provides valuable insights into the cultivation effects and predictive probabilities of different factors as biomarkers.

**Table 2:** Area under the curve (AUC) for the level of cultivation effect.

Level of cultivation effect		Area
Cultivation effect	Low cultivation effect	0.684
	Medium cultivation effect	0.676
	High cultivation effect	0.628

The measures of sensitivity and specificity for the ANN model were presented as an area under the curve (AUC), which represents the entire position of the ROC curve according to the five categories of time spent on the PUBG. Low cultivation = less than 2 h, medium cultivation = between 2–4 h, and high cultivation = more than 4 h.

**Figure 3:**
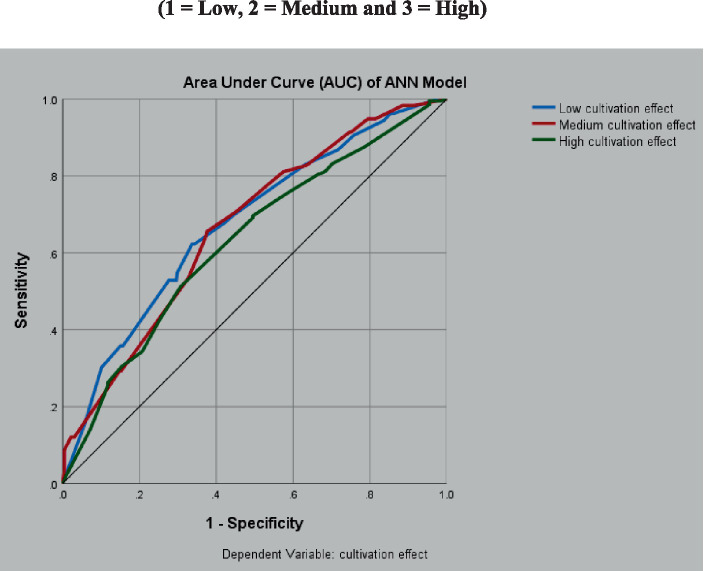
Area under the curve (AUC) of ANN for cultivation effect (1 = Low, 2 = Medium and 3 = High). False positive rate (1-specificity) has been plotted against true positive rate (sensitivity) for all three categories of cultivation effects for ANN model, where the partition for training dataset was 70% and testing dataset was 30%. The initial lambda was chosen to be 0.0000005 and the initial value of sigma = 0.00005. The internal offset is ±0.5. Interpretation of the AUC was based on the Mann–Whitney U-test used for computing the AUC curve.

The data suggest that the high cultivation effect has the highest predictive probability, whereas the low and medium cultivation effects have lower predictive probabilities. To assess the performance of the ANN model, the ROC curve was plotted by measuring the true positive rate (sensitivity) against the false positive rate (1-specificity) at various threshold settings, as shown in Figure 3. The 45-degree line from the upper right angle of the chart to the lower left represents random guessing, and the further away the curve is from this reference line, the better the classification accuracy. The results showed that the AUC for the low cultivation effect is0.684, indicating that the model was quite accurate in predicting this category. In contrast, the AUC for a high cultivation level was 0.628, indicating that the model’s accuracy decreased as the level of cultivation increased. Considering the classification of the model, in the training model, the classification was 77.7%, whereas in the training sample, the classification increased to 80.5%. These results suggesting that the ANN model can be used as an effective tool for predicting the cultivation effect based on selected biomarkers/predictors.

In the context of analyzing this study, it is essential to evaluate the contribution of independent variables in predicting the time spent on PUBG. Table 3 presents the normalized importance of each predictor computed from the ANN model. The results indicated that gaming disorder had the highest normalized importance of 100%, making it the most significant predictor of the cultivation effect. The second most important factor was the religious level, with a normalized importance of 54.6%. The Motivation level was found to be the next significant predictor as well, with a normalized importance of 47.6%. Finally, gaming addiction had the lowest normalized importance among all the predictors, with a value of 44.4%.

**Table 3:** Normalized importance of the independent variables.

Independent variables	Importance	Normalized importance
Age	0.15	16.7%
Education level	0.11	23.2%
Gender	0.051	15.2%
Area of living	0.121	27.2%
Religion level (ARS)	0.222	54.6%
Motivation level (ML)	0.193	47.6%
Gaming addiction (GAS)	0.180	44.4%
Gaming disorder (IGD)	0.406	100.0%

1. The normalized importance was calculated for every predictor based on eight input nodes, one hidden layer that included four units using a tangent hyperbolic activation function, and one output layer with three units. 2. SoftMax was used as an activation function for the output layer, while cross-entropy was computed for the error function, which is equal to 224.111 for training and 104.377 for testing. The training time for the ANN model was estimated to be 00:00:00.44.

**Figure 4:**
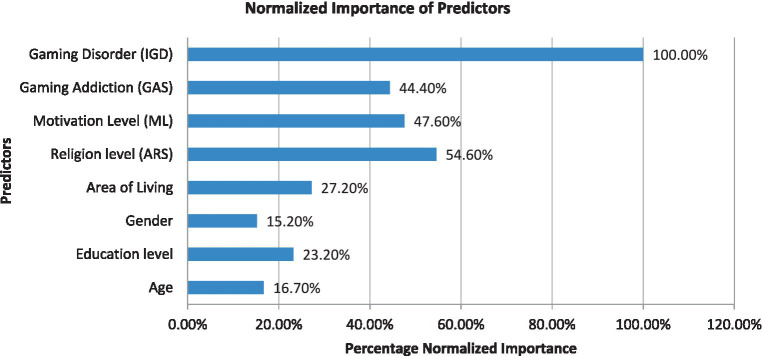
Graphical representation of normalized importance of predictors. The impact of each independent variable in terms of relative and normalized importance, identified in the designed ANN model, is presented with a graphical illustration showing the percentage importance of each predictor (Age, Education Level, Gender, Area of Living, IGD, GAS, ML, and ARS).

Normalized importance can be computed using various methods, such as feature scaling, feature normalization, or feature importance scores, and can be interpreted in different ways depending on the specific neural model and the problem being addressed. To provide a visual representation of each significant indication factor, the figure displays a graphical representation of each predictor’s contribution (%) to the cultivation level. Figure 4 depicts the relative importance of each predictor of the cultivation effect.

## 4 Discussion

The current study specifically examined the gaming behavior of PUBG players, offering a critical analysis and discussion within this particular context. Our findings offer significant insights into the cultivation effect and its association with a variety of factors in the gaming community, thereby setting the stage for a comprehensive discussion on this topic. A notable finding from this research was the correlation observed between gender and cultivation effects. The data revealed a significant disparity in cultivation levels between male and female respondents, with males exhibiting a higher likelihood of possessing a high cultivation level than their female counterparts. This finding is consistent with prior studies that have observed gender disparities in gaming preferences, indicating that males tend to exhibit a greater inclination towards action-oriented games (30, 31). The presence of a gender discrepancy in the cultivation effect observed among PUBG players indicates that societal and cultural influences could potentially contribute to the formation of gaming patterns within this particular demographic. Additional investigations could delve into the fundamental factors that contribute to these disparities between genders, as well as the potential impact of societal norms and expectations.

This study also investigated the level of religiosity as a significant factor. The results revealed a significant inverse correlation between the degree of religiosity and propensity for gaming addiction. These findings align with prior studies that have demonstrated a positive association between religiosity and a reduced tendency towards excessive internet and smartphone usage (32, 33). These findings indicate that individuals who exhibit higher levels of religiosity may be less vulnerable to engaging in problematic gaming behavior. In line with the current study, an Iranian study showed that religious beliefs had a stronger influence than family dynamics, indicating that gamers with stronger religious beliefs were less susceptible to internet gaming (19). This could be because individuals with a higher level of religiosity often have a greater sense of purpose and meaning in life, which can reduce excessive gaming behavior. This study provides valuable insights into the relationship between the duration of PUBG gaming and cultivation effect. The results hold significant implications for individuals who identify as PUBG gamers, as religiosity is an essential component of their personal identity and way of life. Religious beliefs and practices have the potential to act as protective factors against excessive gaming and the associated negative consequences. Religious institutions and communities have the potential to assume a pivotal role in the promotion of responsible gaming habits and in the provision of support to individuals who may be susceptible to developing gaming addiction.

Another significant factor explored in this study was the motivation level. People with a higher level of motivation to play PUBG are more likely to develop gaming disorders. This is in agreement with the uses and gratification theory (34), which suggests that gamers play video games to satisfy psychological and social needs (35). Although motivation level was found to be a significant predictor of excessive gaming behavior, it did not predict the cultivation effect significantly. The complex relationship between motivation and gaming behavior should be further explored in future research.

The present study also examined the correlation between gaming addiction, gaming disorder, and cultivation effect among PUBG players. The results demonstrated significant positive associations among these variables, suggesting that elevated levels of gaming addiction are linked to a heightened probability of developing gaming disorders. This discovery aligns with prior studies that have established a correlation between gaming addiction and gaming disorders (15, 36). However, few studies have documented weak-to-moderate positive interactions between these two factors (36, 37), which may be due to differences in the social outcomes of gaming. The findings of this study indicate that the cultivation effect has the potential to reinforce and sustain problematic gaming behavior. Comprehending these interconnections is imperative for the formulation of effective prevention and intervention approaches that are specifically tailored to address the requirements of individuals engaged in gaming activities.

The employment of an artificial neural network (ANN) model in this research facilitated the anticipation of the cultivation effect by utilizing specific predictors. The findings of this study indicate that gaming disorder emerged as the primary predictor, with religiosity level and motivation level following suit in terms of significance. These findings offer valuable insights into the various factors that contribute to the cultivation effect observed within the community of PUBG players. The classification performance of the ANN model and the normalized importance of predictors indicate that it has significant potential as a valuable tool for predicting and comprehending gaming behavior among individuals in this particular population. This research’s emphasis on PUBG gamers contributes to the body of literature by investigating the patterns of gaming conduct and their resultant effects within a distinct cultural and religious milieu. The results of this study provide valuable insights into the complex nature of gaming addiction and its cultivation effect, specifically within the context of gamers engaged in PUBG. Nevertheless, it is crucial to acknowledge that gamers encompass a wide range of individuals, and that their individual experiences and beliefs may exhibit significant diversity. Subsequent investigations should incorporate an examination of the cultural and contextual elements that could potentially impact the conduct of individuals who engage in gaming activities. This inquiry should encompass an exploration of the divergences observed in various geographical locations, ethnic backgrounds, and religious contexts.

This study exhibits notable strengths, which encompass the implementation of a quantitative research design and the utilization of an artificial neural network (ANN) model for predictive purposes. The study utilized a sample size of considerable magnitude, thereby augmenting the extent to which the findings can be generalized. The implementation of an Artificial Neural Network (ANN) model facilitated the analysis of complex dynamics and their impact on the cultivation effect within players. The classification performance of the model and the normalized importance of predictors yielded valuable insights into the factors that contribute to gaming behavior within this population. Moreover, this research’s emphasis on PUBG players contributes to the extant body of literature by offering a scholarly examination and discourse on the cultivation effect within the framework of culture and religion.

Nevertheless, it is imperative to acknowledge the various constraints. This study utilized a convenient sampling technique, which has the potential to introduce selection bias and restrict the generalizability of the results. Future research endeavors should aim to utilize sampling methods that are more representative in nature with the intention of augmenting the external validity of the findings. Additionally, the research was dependent on self-report assessments, which are susceptible to biases such as recall and social desirability biases. The utilization of objective measures or alternative assessment methods has the potential to enhance the comprehensiveness and reliability of gaming behavior evaluation. Furthermore, the study’s cross-sectional design imposes constraints on making causal inferences, necessitating the inclusion of longitudinal studies to explore the temporal associations between the variables under investigation and cultivation effect. Longitudinal research designs offer a more comprehensive understanding of the dynamic nature of gaming behavior and its long-term consequences. This study primarily concentrated on PUBG, thereby limiting the generalizability of the findings to gamers belonging to different religious or cultural contexts. Subsequent investigations should examine the cultural and contextual variables that potentially influence gaming conduct within heterogeneous populations.

This study provides important insights into the management and prevention of gaming disorders and has numerous implications across various fields. This research confirms the WHO’s classification of gaming illness as a mental health concern by showing the addictive nature of games like PUBG and their influence on daily life (5). This underlines the need for improved awareness and prevention of gaming disorder. Based on these findings, specific intervention strategies should be developed to address psychological issues, such as anxiety, depression, and social withdrawal, which are frequently linked to excessive gaming. The study’s findings on how gaming negatively affects academic performance may lead educational institutions to create policies that accommodate student gamers. Furthermore, this study provides important insights for medical practitioners in identifying and managing health problems associated with extended gaming sessions. Furthermore, this study contributes to the development of culturally appropriate prevention and treatment programs by examining the interaction between individual motives and cultural influences, such as religious beliefs, in gaming behavior. To help legislators create responsible gaming policies and regulations for online gaming platforms, it is imperative that they have a thorough understanding of the effects of gaming behavior. In essence, the study’s multifaceted approach and diverse methodology promulgate both theoretical discussion and real-world applications in areas such as policy formation, healthcare, and education.

## 5 Conclusion

This study makes a valuable contribution to the current literature by investigating the complex interplay between different factors and the cultivation effect observed among players in the PUBG game. The results indicate that the cultivation effect is subject to various influences, including gender, educational attainment, religious affiliation, motivation, gaming addiction, and gaming disorders. The utilization of the ANN model offers a significant asset for forecasting the cultivation effect by leveraging these factors. The findings provide significant insights into the determinants that influence the behavior of PUBG gamers and their subsequent cultivation effect.

The results of this study have significant practical implications for the advancement of preventive strategies and interventions designed to foster responsible gaming behavior and tackle gaming addiction. It is imperative to comprehend the intricate dynamics among variables such as gaming addiction, gaming disorder, motivation, and religiosity to develop efficacious interventions that cater to the unique requirements of individuals engaged in PUBG gaming. To advance our understanding of gaming behavior and facilitate evidence-based interventions, future research should incorporate longitudinal designs, qualitative methodologies, and cross-cultural perspectives. By augmenting our understanding of these domains, we can formulate more holistic approaches to bolster gamers and alleviate the potential adverse ramifications linked to excessive gaming. Future research can delve into the cultural and contextual variables that influence gaming behavior across diverse populations, underscoring the need for a more comprehensive understanding of how various cultural and contextual factors shape individuals’ interactions with video games. This implies recognition of the potential impact of cultural nuances and contextual differences on gaming habits, preferences, and outcomes. Exploring these variables can provide valuable insights into the ways in which diverse communities engage with gaming platforms and their potential effects on their attitudes and behaviors.

## Data availability statement

The raw data supporting the conclusions of this article will be made available by the authors, without undue reservation.

## Ethics statement

The studies involving humans were approved by Social Welfare Department Human Ethical Committee (Approval number 441-WF-12/07/2021). The studies were conducted in accordance with the local legislation and institutional requirements. The participants provided their written informed consent to participate in this study.

## Author contributions

AH: Conceptualization, Data curation, Methodology, Project administration, Validation, Writing – original draft, Writing – review & editing. MS: Investigation, Validation, Writing – original draft, Writing – review & editing. MD: Conceptualization, Data curation, Formal Analysis, Investigation, Methodology, Project administration, Software, Supervision, Validation, Writing – original draft, Writing – review & editing. WH: Formal Analysis, Supervision, Validation, Writing – original draft. SJ: Funding acquisition, Supervision, Validation, Writing – original draft, Writing – review & editing. MK: Conceptualization, Data curation, Formal Analysis, Investigation, Methodology, Project administration, Resources, Software, Supervision, Validation, Visualization, Writing – original draft, Writing – review & editing.

## Appendix 1

Input, hidden, and output layers from ANN model.
